# Evidence for Training-Induced Plasticity in Multisensory Brain Structures: An MEG Study

**DOI:** 10.1371/journal.pone.0036534

**Published:** 2012-05-03

**Authors:** Evangelos Paraskevopoulos, Anja Kuchenbuch, Sibylle C. Herholz, Christo Pantev

**Affiliations:** 1 Institute for Biomagnetism and Biosignalanalysis, University of Münster, Münster, Germany; 2 Montreal Neurological Institute, McGill University, Montreal, Quebec, Canada; McMaster University, Canada

## Abstract

Multisensory learning and resulting neural brain plasticity have recently become a topic of renewed interest in human cognitive neuroscience. Music notation reading is an ideal stimulus to study multisensory learning, as it allows studying the integration of visual, auditory and sensorimotor information processing. The present study aimed at answering whether multisensory learning alters uni-sensory structures, interconnections of uni-sensory structures or specific multisensory areas. In a short-term piano training procedure musically naive subjects were trained to play tone sequences from visually presented patterns in a music notation-like system [Auditory-Visual-Somatosensory group (AVS)], while another group received audio-visual training only that involved viewing the patterns and attentively listening to the recordings of the AVS training sessions [Auditory-Visual group (AV)]. Training-related changes in cortical networks were assessed by pre- and post-training magnetoencephalographic (MEG) recordings of an auditory, a visual and an integrated audio-visual mismatch negativity (MMN). The two groups (AVS and AV) were differently affected by the training. The results suggest that multisensory training alters the function of multisensory structures, and not the uni-sensory ones along with their interconnections, and thus provide an answer to an important question presented by cognitive models of multisensory training.

## Introduction

Early studies concerning sensory learning emphasized the investigation of each modality alone, excluding or manipulating the other senses as interfering variables [1]. Only recently emphasis has been given to multisensory integration and plasticity [2]. Three main models have been proposed as explanation for plasticity induced by multisensory learning [3], [4]: alteration of (a) the uni-sensory structures involved in the multisensory task [5], (b) the interconnection of the uni-sensory structures [6] or (c) the multisensory structures responsible for integrating the stimuli [7].

Music notation reading is an ideal stimulus to study multisensory learning, as it allows studying the integrated processing of visual, auditory and motor information within an established model for experience-induced plasticity [8]. Musicians have an auditory-like representation of written music before they actually hear it [9] and show neurophysiological responses to mismatches between visually presented musical scores and auditorily presented melodies [10]. The presence of differentiated neurophysiological responses to incongruent compared to congruent multisensory stimuli constitutes objective evidence that uni-sensory information have been integrated [11]. Such effects in musicians can be attributed to their long-term training. Furthermore, multimodal musical training is more beneficial for cortical plasticity than unimodal training as has recently been shown by our group [12]. Mismatch Negativity (MMN) is an event-related response to a deviant sound within a stream of standard sounds [13], [14], and it is an established neural marker for detection of incongruences in the auditory perception [15].

The present study aims to answer whether multisensory learning alters multisensory structures or the interconnection of uni-sensory structures. In a short-term piano training procedure musically naive subjects were trained to play tone sequences from visually presented patterns in a music notation-like system [Auditory-Visual-Somatosensory group (AVS)], while training in another group merely involved attentively viewing the patterns, listening to the recordings of AVS group and expressing judgments on the correctness of the recordings [Auditory-Visual group (AV)]. Training-related changes were assessed by pre- and post-training magnetoencephalographic (MEG) recordings of separate auditory, visual and integrated audio-visual MMN responses.

**Figure 1:**
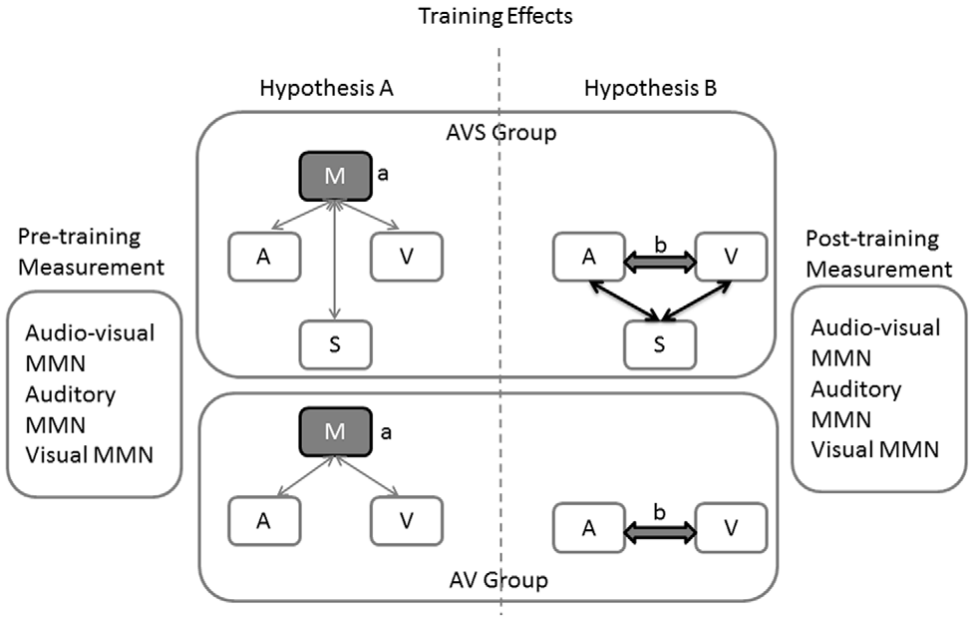
Illustration of the Hypothesis: If multisensory training effects specific multisensory regions (a) the training should affect differently the two groups, hence this region is trained by receiving input from 3 different modalities in the Auditory-Visual-Somatosensory group and 2 in the Audio-Visual one. If training effects the interconnection of the structures (b) the effect should not differ between the groups, hence the trained structure is the same. MEG recordings of an audio-visual an auditory and a visual MMN were conducted pre- and post- training. The bold black lines mark the structures that are trained in each hypothesis. The grey and black shapes mark the structure that contributes to the results of the audio-visual MMN in each hypothesis.

We hypothesize that:

*Hypothesis A:* if the training-induced plasticity altered the integrated audio-visual MMN responses of AVS group in a greater extent than these of AV group, then the training-induced result is probably based on a multisensory structure, since during training this structure receives input from three different modalities in AVS group but only from two in AV group (c.f. (a) Fig. 1, left side).

*Hypothesis B:* if the resulting plasticity altered the audio-visual responses of the AVS and the AV groups in an equal degree, then the training-induced result would be based on the interconnection of auditory and visual modality. That is because during the training the interconnection of auditory and visual structures receives input only from those two modalities, which are trained in both groups. The role of the uni-sensory structures in the resulting training-induced plasticity was controlled by uni-sensory MEG recordings of auditory and visual MMN.

## Materials and Methods

### Subjects

Twenty four individuals participated in the experiment (mean age = 25.86; SD = 3.17; 8 males) and were equally and randomly divided into two groups: an auditory-visual-sensorimotor (AVS) and an auditory-visual (AV) group. None of the subjects had received any musical education apart from the compulsory lessons in school prior to participating in the study. All subjects were right handed according to the Edinburgh Handedness Inventory [16], and had normal hearing as evaluated by clinical audiometry. Subjects provided written consent prior to their participation in the study. The study protocol was approved by the ethics committee of the medical faculty of the University of Münster and the study was conducted according to the Declaration of Helsinki.

### Stimuli

Stimuli were prepared for three different conditions in the MEG recordings that are described in more detail below: an audio-visual condition, an auditory control condition, and a visual control condition.

**Figure 2:**
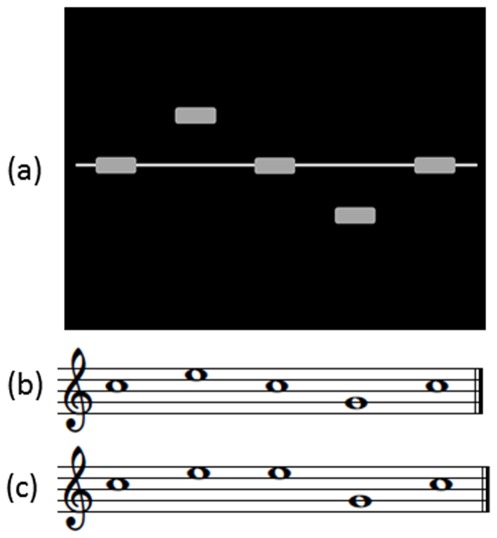
Illustration of one of the audio-visual stimuli. The upper panel (a) shows the visual part of the stimulus while the middle one (b) the musical notation of a congruent auditory part of the stimulus and the lower one (c) the musical notation of an incongruent auditory part. The incongruency violated the presented visual pattern in terms of contour and thus for this tone it did not match the corresponding bar in the visual stimulus. This position of the incongruent bar-tone pair was never the first or the last one and was counterbalanced across and within the positions two, three and four of the patterns. The auditory part of the stimulus was presented 1 sec after the visual.

The visual part of the stimuli constructed for the audio-visual condition consisted of five short horizontal bars that were presented simultaneously side by side, but at different heights along the vertical axis, spaced apart by the same length as the bars. They were either in a low, middle or high position on the screen. A thinner horizontal line at the height of the middle bar that spanned the whole width of the screen was presented as a visual anchor for the middle position (c.f. Figure 2). Six different patterns were prepared. For each visual pattern, the first and the last bars were always in the middle position. The auditory part of the stimuli consisted of short melodies or tone patterns composed of five tones that each had one of three different pitches (G4, C5, E5). Auditory stimuli were generated by means of a digital audio workstation (Steinberg, Cubase SE 3.0.3.658; http://www.steinberg.net) in grand piano timbre. Duration of the tones was 300 ms including 10 ms rise and fall, with a inter tone interval of 200 ms. In the audio-visual condition, visual and auditory stimuli were always presented together, but the auditory part of the stimuli commenced 1 sec after the visual. Auditory and visual stimuli were combined so that they matched regarding a simple rule: Each piano tone corresponded to a specific position of the bars along the vertical axis in the visual stimuli: C5 to the middle position, E5 to the higher one and G4 to the lower one. In congruent audio-visual stimuli all piano tones corresponded correctly to the visual pattern, when the pattern was ‘read’ from left to right as in Western writing. In incongruent stimuli one of the tones did not match the corresponding bar in the visual stimulus. This incongruent bar-tone pair was never at the first or the last position in time (and correspondingly on the horizontal axis). Also, the incongruency violated the presented visual pattern in terms of contour and not simply in terms of a different tonal interval. Six different incongruent stimuli were presented to the subjects, each corresponding to a congruent one. Moreover, the incongruency was counterbalanced across and within the positions two, three and four of the patterns.

For the auditory control condition, an auditory oddball paradigm was used: Two of the piano tones used in the audio-visual stimuli (G4 and C5) were used as standard and deviant tones. Assignment of either tone as standard or deviant was counterbalanced across subjects. Deviant tones were presented at a probability of 0.2 with the constraint that at least three standard tones were presented between any two deviants. The inter stimulus interval varied randomly between 450 and 750 ms. The visual control condition was set up in analogous fashion, as the visual oddball paradigm. The stimuli consisted of one fixation cross in the middle of the screen along with one short horizontal bar presented at equal distance either above or below the cross. The position of the bar differed between standards and deviants only in terms of height on the vertical axis. Use of the high or low bars as standard or deviant was counterbalanced across subjects. They were presented for 400 ms, and the inter stimulus interval varied randomly between 400 and 600 ms; the fixation cross was presented continuously.

The stimuli used in the training procedures were similar to the visual part of the stimuli used in the audio-visual condition. Specifically, three out of the six patterns used were identical with the ones presented during the MEG recording and three patterns were new but constructed along the same principles. Correspondingly, three of the six patterns used in the MEG recordings were presented during training and three were not used for training.

### Design

#### MEG recordings

Both pre- and post-training MEG recordings were identical. Magnetic fields were recorded with a 275 channel whole-head system (OMEGA, CTF Systems Inc, Port Coquitlam, Canada) in a magnetically and acoustically shielded room. Data were acquired continuously during each presentation block with a sampling rate of 600 Hz. Subjects were seated upright, and their head position was comfortably supported with small pads inside the dewar. The subject's alertness and compliance were verified by video monitoring. The auditory stimuli were delivered via 60 cm long silicon plastic tubes at 60 dB SL above the individual hearing threshold that was determined with an accuracy of at least 5 dB at the beginning of each MEG session for each ear. The visual stimuli were presented on a flat panel display (LG 1970 HR) located approximately 150 cm away from the subject's nasion. The monitor was run at 60 Hz and a spatial resolution of 1280×1024 pixels. The viewing angle of the stimuli ranged from −3.86° to 3.86° in the horizontal direction and from −1.25° to 1.25° in the vertical direction. Each session consisted of three conditions: an audio-visual, a visual and an auditory. The audio-visual condition consisted of two runs, lasting approximately 14.5 min each. The six different audio-visual patterns were randomly combined to build one run containing 150 stimuli, 75 of them congruent and 75 incongruent. The recording was synchronized to the presentation of all tones of the congruent trials, whereas it was synchronized only to the incongruent tone of the incongruent trials. This resulted in an incongruent to congruent ratio of 20%. Subjects had to indicate after each presented audio-visual stimulus, and within 1.5 sec of the last heard note, if the auditory presented melody was congruent or incongruent with the visually presented pattern according to the rule “the higher the position of the bar, the higher the pitch”. The responses were given via button presses. The visual condition consisted of one run of the visual oddball paradigm, lasting approximately 14.5 min and including 850 standard and 150 deviant stimuli. Subjects were instructed to direct their gaze to the fixation cross but to pay attention to an audiobook presented via the plastic tubes. The auditory condition consisted of one run of the frequency oddball paradigm including 850 standard and 150 deviant stimuli, lasting approximately 14.5 min. During the auditory oddball paradigm subjects were instructed not to pay attention to the sound stimuli and watched a soundless movie of their own choice.

#### Training

Eight sessions of training took place during a period of 10 days over weeks. The first training was done after the pre-training MEG recording and the last immediately before the post-training recording. Subjects were seated in front of a digital piano while a screen adjusted approximately at the height of the nasion presented the visual patterns. The six visual patterns were pseudo-randomly combined in one run consisting of 150 stimuli presented 10 sec each. One training session lasted 25 min. Each time a new pattern was presented a notification sound informed the subject that the pattern had changed. The AVS group's task was to play the corresponding pattern to the piano during the 10 sec the pattern was presented by using three keys (G4, C5 and E5) assigned always to the same fingers (thumb of left hand for G4, thumb of right hand for C5 and middle finger of right hand for E5). The responses of the AVS group were recorded via MIDI. Each subject of the AV group listened to all of the training sessions of one randomly assigned subject from the AVS group. The AV group's task was to listen to the recordings of the AVS group while seeing the same pseudo-random sequence of patterns presented to the AVS group and press the right- or left-foot pedal of the piano after each pattern to indicate that the melody they heard was congruent with the visual pattern or not. This task was chosen to ensure that the AV group paid attention to the stimuli although they were not engaged in active playing.

### Data analysis

The Brain Electrical Source Analysis software (BESA research, version 5.3.7, Megis Software, Heidelberg, Germany) was used for the processing of the MEG data. The recorded data were separated in epochs of 600 ms including a pre-stimulus 200 ms interval. Epochs containing signals larger than 2.5 pT were considered artifact contaminated and excluded from the averaging. Data were filtered offline with a high pass filter of 1 Hz, a low pass of 30 Hz and an additional notch filter at 50 Hz. Epochs were baseline corrected using the pre-stimulus interval from −100 to 0 ms. Averages were computed separately for the congruent and the incongruent stimuli of the audio-visual condition and for the standards and the deviants for the auditory and visual conditions. The difference responses were computed by subtracting the averaged responses of the congruent from those of the incongruent stimuli for the audio-visual condition and the averaged responses of the standard from the deviant for the auditory and visual condition.

Current density reconstructions (CDR) were calculated on the difference responses for each subject using the LORETA method [17]. LORETA directly computes a current distribution throughout the full brain volume instead of a limited number of dipolar point sources or a distribution restricted on the surface of the cortex and provides a solution for the inverse problem based on the smoothness of all possible activity distributions for explaining the data. This method has been used successfully previously for the mapping of MMN [18], [19] and has advantages for a paradigm as the one used in the present study. Specifically, in this paradigm one cannot a priori exclude the possibility that more than one sources respond in a temporally correlated form. Therefore, it is more appropriate to use a method that can reconstruct the entire grey matter volume without a priori assumptions for the number of activated sources. A separate time window of 10 ms for each condition was used for the CDR. For the determination of the appropriate time windows the following procedure was used: The sensor data of the responses to the standard condition were subtracted from the ones of the deviant condition and thus the difference responses were obtained. The grand average global field powers of the difference responses were calculated for the pre- and post- training MEG recordings. The appropriate MMN time windows were defined as the peak of the grand average global field powers of the difference responses within the time range of 120–250 ms (a typical latency range for MMN [20]). A time window of 10 ms for each condition was used including both pre- and post-training peaks (i.e. 180–190 ms for the audio-visual condition; 134–144 ms for the auditory condition and 120–130 ms for the visual condition). Each individual's mean image over the selected time-window was calculated and projected onto a standard MRI template based on the Montreal Neurological Institute (MNI) template. Smoothing was done by convolving an isotropic Gaussian kernel with 7 mm full width half-maximum (FWHM) through Besa's smoothing utility.

Statistical Parametric Mapping 8 (SPM8, http://www.fil.ion.ucl.ac.uk/spm) was used for the statistical analysis of the CDRs. Specifically, a separate Flexible Factorial Model was designed for each condition (audio-visual, auditory and visual) including the factors subject (to control for the repeated measures), MEG recording (pre- and post-training) and group (AVS and AV). This model is SPM's equivalent to a 2×2 mixed model ANOVA with between subjects factor group (AVS and AV) and within subject factor MEG recording (pre- and post- training). Results were then constrained in gray matter using a mask, thereby keeping the search volume small and in a physiologically reasonable source. A permutation method for peak - cluster level error correction (AlphaSim) at 1% level was applied for this whole head analysis, as implemented in REST software [21], by taking into account the significance of the peak voxel (threshold *p*<0.001 uncorrected) along with the cluster size (threshold size >84 voxels), thereby controlling for multiple comparisons. Visualization was done using MRIcron (http://www.mccauslandcenter.sc.edu/mricro/mricron/).

Additionally, in order to confirm the validity of the MMN response one-sample *t*-tests were applied on the CDR's of the responses of all subjects separately for each condition. For this analysis global normalization was applied as implemented in SPM8, in order to distinguish between global and regional activity. This method is necessary in order to apply a one-sample *t*-test in CDRs [22], [23]. Except when otherwise noted, results were corrected with AlphaSim error correction at 1% level (voxel threshold *p*<0.001; cluster size >84 voxels).

## Results

### Behavioral responses

#### Pre- vs post- training testing comparison

The discriminability index d′ was calculated for pre- and post-training testing and entered in a 2×2 mixed model ANOVA with factors testing (pre- and post-training) and group (AVS and AV). The ANOVA results did not reach significance neither for the main effects of group and testing nor for their interaction implying, thus, that no differences between the two groups were observed in the behavioral responses.

#### Pre-training testing

As expected, the results of the behavioral discrimination task in the audio-visual condition (button pressings on whether the presented trial was congruent or incongruent) showed a high level of correct responses already in the pre-training testing (mean correct responses: 273.21, *SD* = 36.07; representing 91.07%).

#### Training performance

In order to investigate the training performance of the AVS group the mean of correct sequences of the first and the last session, along with two categories of mistakes (replacement of note(s) and omission of note(s)) were taken into account. Subjects reached a high performance on the amount of correct responses already from the first training session [mean of correct sequences = 145.11 (96.74%) *SD* = 3.65; mistakes: omission of note(s) = 1.22 (0.81%) *SD* = 1.48; incorrect note(s) = 3.66 (2.44%) *SD* = 2.39]. Last session's performance [mean of correct sequences = 148.25 (98.83%) *SD* = 2.49; mistakes: omission of note(s) = 0.166 (0.1%) *SD* = 0.38; incorrect note(s) = 1.58 (1.05%) *SD* = 2.35] was compared with the first one's using a paired sample t-test, and revealed a significant improvement on the accuracy of performing the sequences on the piano [mean of correct sequencies: *t*(11) = 3.97; *p*<0.05; omission of note(s): *t*(11) = 3.52; *p*<0.05; incorrect notes(s): *t*(11) = 2.49; *p*<0.05].

### MEG results

#### Audio-visual condition

**Figure 3:**
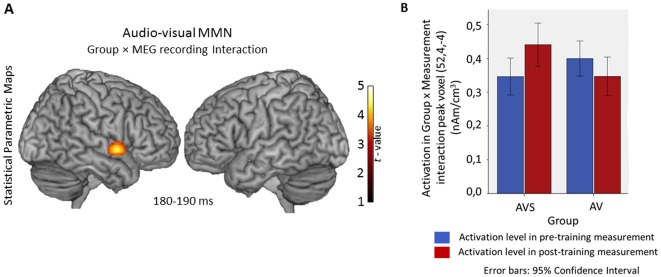
A: Rendering of the Statistical Parametric Maps of the interaction effects of Group×MEG recording in the audio-visual condition. Location of the Group×MEG recording interaction effect in the audio-visual condition: Right Superior Temporal Gyrus, BA 22;. Threshold was AlphaSim corrected at *p*<0.001 by tanking in to account the voxel peak significance (threshold *p*<0.001 uncorrected) along with the cluster size (threshold size >84 voxels). B: Bar plot of the activation in the peak voxel identified by the Group×MEG Recording interaction for each group in pre- and post-training recording. Error bars show 95% Confidence interval.

Pre- vs post- training comparison: The statistical analysis of the audio-visual MMN maps (Figure 3) revealed a significant interaction of group×MEG recordings thereby showing a differentiated effect of training between the two groups. This interaction was investigated using a t-contrast to examine the specific direction of the effect. The result was located in one cluster covering a region of STG, BA 22 (peak coordinates: x = 52, y = 4, z = −4; t(22) = 4.38; cluster size = 425 voxels; p<0.001 AlphaSim corrected) revealing that this region was more affected by the training in AVS than in the AV group. The main effect of MEG recording failed to reach significance. Subsequent analyses of paired sample t-test between the pre- and post-training recordings for each group revealed that the AVS group significantly increased activation in a cluster covering a region of STG, BA 22 (peak coordinates: x = 49, y = 2, z = 2; t(11) = 4.71; cluster size = 135; p<0.001 AlphaSim corrected) while the AV group showed no significant difference between the pre- and post-training recording. Figure 3 presents the statistical parametric map of the group×MEG recording interaction found in the audio-visual condition and the activation of the peak voxel of this interaction separately for both groups and both recordings. All anatomical regions are defined in Talairach space using TalairachClient (http://www.talairach.org/) after the transformation of SPM's MNI coordinates in Talairach space using icbm2tal (http://brainmap.org/icbm2tal/).

**Figure 4:**
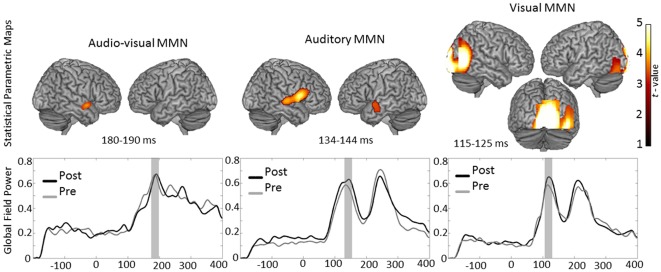
Rendering of the Statistical Parametric Maps of the generators of audio-visual, auditory and visual MMN as revealed by the one sample t-test analysis. Threshold was common for all three analyses: AlphaSim corrected at *p*<0.001 tanking in to account the voxel peak significance (threshold *p*<0.001 uncorrected) along with the cluster size (threshold size >84 voxels). Location of the peak voxel in audio-visual MMN: right Superior Temporal Gyrus (STG), BA 22; location of the peaks in auditory MMN: right STG, BA 22; right Postcentral Gyrus BA 44; left Middle Temporal Gyrus BA 21; locations of the peak voxel in visual MMN: Right Lingual Gyrus, BA 17 and Right Lingual Gyrus, BA 18. Lower Panel shows the grand average global field power of pre- and post- training MEG recording for each condition. The time interval of choice for each condition is marked grey.

MMN generators: For the investigation of the audio-visual MMN generators the images of the pre-training MEG recording of both groups were entered in the one sample t-test, since all subjects were musically naive prior to the training. Results of the MMN generators of audio-visual condition revealed one cluster of activity located in the inferior part of the right Superior Temporal Gyrus (STG), Brodmann Area (BA) 22 (peak coordinates: x = 54, y = 4, z = −10; t(22) = 3.86; cluster size = 399 voxels; p<0.001 AlphaSim corrected). The statistical map of the audio-visual MMN generators is presented in figure 4.

#### Auditory condition

Pre- vs post- training comparison: The results of the auditory MMN condition indicated that the two types of training did not affect the uni-sensory MMN responses differently. Specifically, the statistical analysis for auditory condition revealed that neither the main effect of MEG recording nor the interaction of group×recording reached significance, even when the peak threshold was lowered at the level of p<0.005.

MMN generators: Since the images of the auditory condition did not reveal a significant MEG recording effect nor a MEG recording×group interaction, they were all entered in the MMN generator one sample t-test, in order for the best possible signal to noise ratio to be achieved. MMN sources for the auditory condition were located bilaterally in auditory areas. One cluster including two peaks was located on the right hemisphere in the STG, BA 22 (peak coordinates: x = 64, y = 6, z = 2; t(46) = 4.18; cluster size = 2624 voxels; p<0.001 AlphaSim corrected), along with a peak at the Postcentral Gyrus (PCG) (peak coordinates: x = 54, y = 10, z = 10; T = 4.61; cluster size = 2624 voxels; p<0.001 AlphaSim corrected); while the second cluster was located in the left Middle Temporal Gyrus, BA 21 (peak coordinates: x = −56, y = 0, z = −8; t(46) = 3.48; cluster size = 1032 voxels; p<0.001 AlphaSim corrected). The statistical map of the auditory MMN generators is presented in figure 4.

#### Visual Condition

Pre- vs post- training comparison: As for the auditory condition, the results of the visual MMN condition indicated that the two types of training did not affect this modality's MMN responses differently. Specifically, the statistical analysis for the visual condition revealed that neither the main effect of MEG recording nor the interaction of group×recording reached significance, even when the peak threshold was lowered at the level of p<0.005.

MMN generators: As with the auditory condition, since the images of the visual condition did not reveal a significant MEG recording effect nor a MEG recording×group interaction, they were all entered in the MMN generator one sample t-test. MMN generators of the visual condition were located in one extended cluster covering a broad region in the occipital cortex including two peaks: one at the right Lingual Gyrus, BA 17 (peak coordinates: x = 4, y = −95, z = −2; t(46) = 4.97; cluster size = 8560 voxels; p<0.001 AlphaSim corrected), and one at the right Lingual Gyrus, BA 18 (peak coordinates: x = 38, y = −72, z = −6; t(46) = 3.82). The statistical map of the visual MMN generators is presented in figure 4.

#### Effects in the vicinity of P2

**Figure 5:**
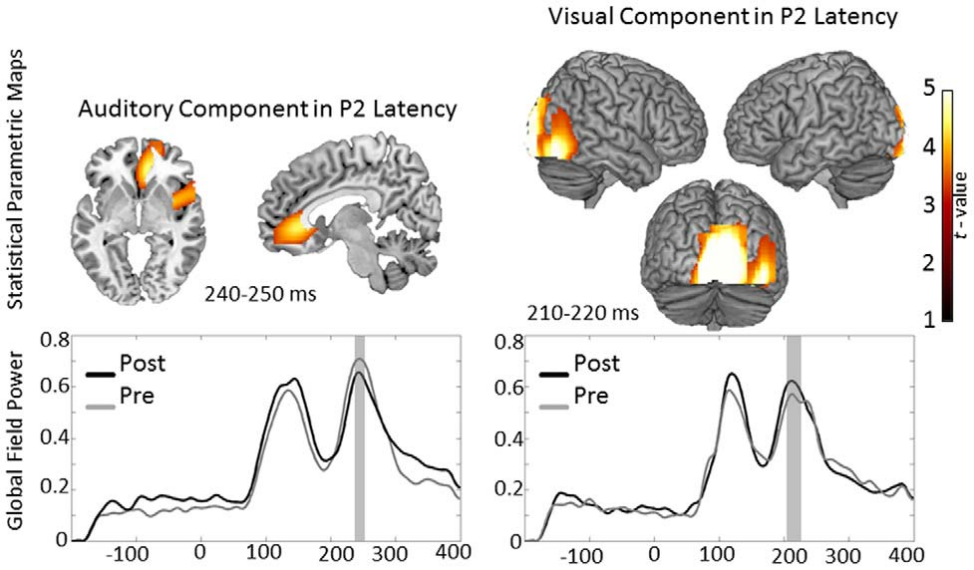
Sagittal and axial view of the statistical Parametric Maps of the peaks found in auditory condition and rendering of the Statistical Parametric Maps of the peaks found in the visual condition in the latency of P2. Location of the activation in the auditory condition: right Insula, BA 13; right Anterior Cingulate Cortex, BA 24. Location of the activation in the visual condition: right Lingual Gyrus, BA 18. Threshold was AlphaSim corrected at *p*<0.001 by tanking in to account the voxel peak significance (threshold *p*<0.001 uncorrected) along with the cluster size (threshold size >84 voxels). Lower Panel shows the grand average global field power of pre- and post-training MEG recording for each condition. The time interval of choice for each condition is marked grey.

An additional finding of the present study was that the difference responses in the uni-sensory conditions revealed another clear peak in the grand average global field power around the latency of P2 (i.e. 240–250 ms for the auditory condition and 210–220 ms for the visual condition; Figure 5). In order to determine if this later differential response to deviants compared to standards was affected by training, data at the latency of these peaks were analyzed according to the same procedure that was used for the MMN. The one sample t-test of this peak in the auditory condition revealed two clusters of activity in the right hemisphere: one located at the Insula BA 13 (peak coordinates: x = 36, y = 14, z = 8; t(46) = 4.95; cluster size = 5199 voxels; p<0.001 AlphaSim corrected) and one located at the Anterior Cingulate Cortex BA 24 (peak coordinates: x = 8, y = 38, z = −4; t(46) = 4.52; cluster size = 1460 voxels; p<0.001 AlphaSim corrected). The one sample t-test of this peak in the visual condition revealed one cluster located in the Occipital Lobe with one peak in the Lingual Gyrus, BA 18 (peak coordinates: x = 6, y = −98, z = 8; t(46) = 4.81; cluster size = 12933 voxels; p<0.001 AlphaSim corrected) and one in the Lingual Gyrus, BA 17 (peak coordinates: x = −8, y = −100, z = −14; t(46) = 4.01; cluster size = 12933 voxels; p<0.001 AlphaSim corrected). The separate Flexible Factorial Model analyses both for the auditory and visual condition revealed that neither the main effect of MEG recording nor the interaction of group×MEG recording reached significance.

## Discussion

The goal of this study was to answer whether plasticity induced by multisensory training affects functionally *genuine multisensory modules*, uni-sensory ones, or the *interconnection of uni-sensory structures*. We compared the effects of two multimodal training paradigms on the processing of uni- and multimodal stimuli using MEG. Training involved learning to play short melodies on the piano from a music notation-like visualization (AVS group) or reading the same visualization while listening to the recordings of the other group (AV group). Thus, both trainings involved the modalities that were tested to exactly the same extent, with the difference that the AVS training additionally involved the sensorimotor modality. The experimental results confirmed the hypothesis that the two groups (AVS and AV) were affected differently by the training and thus revealed new insights on the mechanisms of multisensory learning.

The behavioral results indicated that the task was easy for the participants. This is not surprising since the initial reason that this testing was introduced was to ensure that subjects paid attention to the stimuli and a ceiling effect already in the pre- training testing was expected. Moreover, the necessary delay in the response (so that the finger movement does not affect the MEG data) may have eliminated possible differences based on reaction times.

It must be noted that training's intention was solely to engage all 3 modalities (for the AVS group – or both modalities for the AV group) to a task relevant to this simplified music reading paradigm. Since the task of the AV group's training (identifying the errors) was not difficult (as the behavioral data of the pre-training measurement already revealed), also the task of the AVS group had to be similarly easy. That was the reason that only 3 different keys-fingers were used and 10 seconds during which participants had to perform each sequence. This resulted in a fairly high performance already in the first training session (96.74%). Nevertheless the statistical analysis of the trainings revealed a small but significant improvement in performing the sequences on the piano. The engagement of the 3 modalities to the task has proven sufficient to reveal significant effects in the audio-visual MMN and at the same time preserved a similar level of difficulty (or ease) for both groups of training.

The generators of the auditory MMN were found bilaterally in the temporal cortex but with a higher amplitude and a larger cluster in the right hemisphere than the left one confirming, thus, previous results regarding musical stimuli [24]. The PCG activation (BA 44) that was found in the right hemisphere can be attributed to an automatic attraction of the attention by the deviant stimulus, along with the use of working memory during the discrimination process [25]. The audio-visual MMN was generated by the right STG confirming previous results obtained with equivalent current dipoles approach [15], [26]. The fact that the left temporal cortex did not exceed the threshold of significance can be attributed to the typical right lateralization of the auditory MMN with respect to musical stimuli [27].

The visual MMN is an interesting finding since to our knowledge there is only one previous study using MEG that was able to demonstrate a visual MMN [28]. The experimental paradigm of the Urakawa et al. (2010) study induced a change in the periphery of the visual field by alternating the color of a series of LEDs surrounding a screen that presented a movie. In our paradigm the stimuli were in the center of the visual field and the change generating the MMN was the location of the presented bar. The fact that the standard and deviant stimuli were counterbalanced across the subjects argues that the response in the group level is unlikely be due to the firing of different set of neurons responding to different positions of the visual field. Moreover the attention in the paradigm used in our study was guided to the auditory modality (listening to an audiobook) while the attention in the Urakawa et al. study was guided to the visual input. This finding suggests that the visual MMN shares the pre-attentive attributes of the auditory one. However, further research seems to be necessary in order to reveal more details for this visual response.

The training effects on the audio-visual MMN represent the main finding of this study. Results revealed that the auditory-visual-somatosensory training was more beneficial for the resulting plasticity than the auditory-visual one. This finding suggests that the trained module was functionally affected by all three different modalities. As presented in our hypothesis (Fig. 1) this effect can influence only a functionally multisensory region, since if the training affected the interconnection of the uni-sensory regions the audio-visual MMN would not be differently affected by the two types of training. Moreover, if the training affected the uni-sensory structures and their interconnection as well, the auditory MMN would be differently affected by the two types of training. This result is contributing to a long standing question regarding the resulting plasticity of the multisensory training as noted by other studies [3], [4]. Moreover this finding is supported by recent studies regarding multisensory attributes of cortical structures [29] revealing that a large part of the neocortex responds to multisensory stimuli [2], even within the areas A1 [30] and V1 [31]. Furthermore the STG, where the effect of multisensory training was found, is generally considered as a multisensory structure [2]. The absence of a main effect of MEG recording can be attributed to the fact that the training of the AV group was not that demanding since it was a similar procedure as the one used for the behavioral testing that reached a ceiling effect.

Another important finding of the present study is that the audio-visual MMN was found to be delayed in comparison to the uni-sensory ones. A possible interpretation for this is that the latency of the effect might indicate the cognitive load [24], and thus the audio-visual response having to integrate the visual and the auditory input takes longer to be processed.

Recent studies of our group [12], [32] suggested that multisensory training resulted in greater effects on the auditory MMN than uni-sensory. Based on the results of this study one would expect that AVS group of the present study would be more affected than AV group in the auditory condition. This was not the case. Auditory MMN was not affected by training neither a group difference was detected. This result can be attributed in the different paradigm used for measuring MMN, since Lappe et al. (2008; 2011) used a more difficult three- and a six- tone pattern to measure MMN, whereas in the present study only a frequency MMN was used. These two different paradigms are thought to underlie different processes [33] and are differently affected by musical training. Specifically, the frequency MMN seems not to be affected by musical training [34] while the pattern MMN does [35]. An additional difference is that the training in the previous study focused on learning to play a specific musical progression while the training in the present study focused on simple 5 tone patterns.

The auditory oddball paradigm used as a control condition is simpler than the auditory part of the audiovisual condition. Nevertheless, it still accounts for testing alterations of the reactivity of the auditory cortex, it has been widely used as such in the literature [36] and there are specific reasons why it was judged as an appropriate control condition for this paradigm: In the audio-visual condition an incongruency seems plausible to affect pitch height discrimination and its mapping to the visual representation. The underlying auditory process that supports the identification of the incongruency seems to be the comparison of each individual tone's height with the expected one. In other words, for each single tone the auditory cortex expects a specific pitch, while another one of a different height is presented. The generation of this expectancy in the audio-visual condition is based on the visual input, while in the auditory condition (oddball paradigm) from the memory trace of the preceding tones, but the comparison process is the same. This was the reason that a simple pitch height oddball paradigm was judged as an appropriate control condition.

Indeed, if there was a way to identify the sequences as a complete auditory object without generating a specific pitch height expectancy for each individual tone, a pattern MMN would be more appropriate. However, the structure of the stimuli used in the study contradicts the possibility that the sequences were learned in such a way: During testing (both pre- and post- training) subjects were exposed to 6 different sequences that all started from the same note and continued without following a specific rule (such as all notes going up or all notes going down). This caused a necessity of “reading” all notes of a sequence in order for an auditory expectancy for each single tone to be build. Moreover, during training 3 of the 6 sequences used were new and only used during the training (they were not presented during pre- or post- training testing). Consequently, a kind of learning of the specific sequences used in testing that would result in the lack of the need for a tone-by-tone expectancy does not seem plausible.

The behavior of the visual MMN replicated the results of the auditory one: a training effect was not detected. This finding seems to be reasonable if one considers the fact that the training focused on the auditory expectancy produced by the visual input and not on the visual input itself. Moreover this result, along with the results of the auditory condition, indicates that the uni-sensory structures were not affected by the applied multisensory training while the multisensory one did. Of course, the absence of a significant effect cannot be a conclusive proof; however, the design of the study is similar to the one used in previous relevant studies concerning uni-sensory auditory plasticity [12], [32] (except for the use of a frequency MMN as a control condition that has been discussed above) and in these studies group differences were revealed. Thus, the absence of a significant group effect in the uni-sensory modalities could not simply be attributed to the study's design or alternatively to a lack of statistical power, as a significant group×MEG recording effect was found in the audio-visual condition, where the signal to noise ratio (SNR) can be assumed to be comparable.

An additional finding of the present study was the peak of the uni-sensory structure's activity found in the difference global field power in the latency of P2. This activation can be attributed to an automated attraction of attention by the deviant stimuli. This interpretation is supported by the locations of the activity, which are directly connected with attention, both for the auditory condition (i.e. ACC, Insula) [37], [38] as well as for the visual one (i.e. Cuneus) [39]. The fact that this response was not affected by training indicates that mere attentional sources should not have a significant influence on the group×MEG recording interaction that was found in the audio-visual condition.

### Conclusion

The findings of the present study argue that plasticity due to short-term multisensory training alters the function of separate multisensory structures, and not the uni-sensory ones along with their interconnection. This result contributes to an important question presented by cognitive models of multisensory training. Moreover several questions regarding the effects of multisensory training on the uni-sensory MMN are generated.
