# Correction: Evidence for Training-Induced Plasticity in Multisensory Brain Structures: An MEG Study

**DOI:** 10.1371/annotation/6faed8de-2067-4913-bd8a-f87151cc74c1

**Published:** 2012-05-08

**Authors:** Evangelos Paraskevopoulos, Anja Kuchenbuch, Sibylle C. Herholz, Christo Pantev

There was an error in the corresponding author designation. Christo Pantev is the corresponding author. The published email address is correct. 

